# The National Relief Fund in Scotland

**Published:** 1916-01-22

**Authors:** 


### THE NATIONAL RELIEF FUND IN SCOTLAND.

The Report of the Scottish Advisory Committee on the Administration of the National Relief Fund in Scotland, recently issued as a White Paper, does much more than account for the £26,000 expended up to March 31 under its direction. It is a document throwing much light on industrial conditions in the Northern part of the kingdom, and is to be judged in its relation to the future rather than as a record of work accomplished.

The Committee consisted originally of twenty-three members, of whom two were women; and subsequent changes in its composition increased the women members to four. Cases of distress arising out of the calling-up of Reservists and of enlistment were relegated to the Soldiers' and Sailors' Families' Association, and there remained for the Central Committee to deal with distress caused by dislocation of industry resulting from the war. Local committees were set up in every country district and in every burgh with a population of 7,000 or over. These were supplemented by smaller local sub-committees, and the services of nine inspectors were lent by the Local Government Board and the National Health Insurance Commission in order to organise the voluntary workers.

So far civil distress in Scotland resulting from the war has been much less than was anticipated. The occupations most seriously and durably affected were fishing, with its subsidiary industries, and the letting of lodgings. Relief in selected cases was given in accordance with the scale drawn up by the Government Committee for the Prevention and Relief of Distress, and power was given last March to local committees to supplement the allowances granted under this scale to the extent of 15 per cent., owing to the rise in prices. Direct assistance was granted under these conditions to the extent of £21,345 among a total of ninety-four counties, districts, and burghs.

The problems of distress among women present special difficulties, and a Scottish Committee on Women's Employment was constituted in September 1914 to deal with them. It is greatly to be deplored that no profitable market has so far been found for more than a very limited amount of hand-knit and hand-woven articles produced in the Highland districts. The impotence of voluntary effort to secure anything like a satisfactory demand for home industries of the type fostered by the Scottish Women's Committee is the salient feature in this part of the Report. The twenty-three workshops opened in various localities are doing excellent work, though the numbers employed in them began to decrease towards the end of March. No doubt the experiment has undergone expansion during the present winter. But attempts to graft new occupations or even to encourage and develop former industries among the people meet with very moderate success. What seems to be needed is a group of men and women possessed of practical commercial experience, fired by philanthropic enthusiasm. There ought to be some way of directing home industries in such a manner that a large public demand is created, the fructifying streams of which can be secured for the home workers instead of being diverted to feed the capitalist. Meantime the experience accumulated in the administration of funds such as the above is beginning to build up a basis for future operations.

